# Histological treatment response to neoadjuvant chemotherapy predicts the survival outcomes of patients with resectable pancreatic cancer: validation of the Japan Pancreas Society grading system

**DOI:** 10.1007/s00595-026-03325-w

**Published:** 2026-06-23

**Authors:** Daisuke Douchi, Mika Ando, Yuichiro Umino, Hideaki Sato, Mitsuhiro Shimura, Koetsu Inoue, Masahiro Iseki, Shuichi Aoki, Keigo Murakami, Takayuki Miura, Shimpei Maeda, Masaharu Ishida, Masamichi Mizuma, Atsushi Masamune, Toru Furukawa, Michiaki Unno

**Affiliations:** 1 Department of Surgery, Tohoku University Graduate School of Medicine, 1-1 Seiryo-machi, Aoba-ku, Sendai, Miyagi 980-8574 Japan; 2 Department of Investigative Pathology, Tohoku University Graduate School of Medicine, 2-1 Seiryo-machi, Aoba-ku, Sendai, Miyagi 980-8575 Japan; 3 Division of Gastroenterology, Tohoku University Graduate School of Medicine, 1-1 Seiryo-machi, Aoba-ku, Sendai, Miyagi 980-8574 Japan

**Keywords:** Pancreatic ductal adenocarcinoma, Neoadjuvant chemotherapy, Histological treatment response, Prognosis, Japan Pancreas Society grading

## Abstract

**Purpose:**

To validate the prognostic value of histological treatment response, assessed using the Japan Pancreas Society (JPS) grading system, in patients with resectable pancreatic ductal adenocarcinoma (PDAC) treated with neoadjuvant chemotherapy (NAC) followed by surgical resection.

**Methods:**

We analyzed, retrospectively, data from 117 consecutive patients who underwent NAC and curative-intent PDAC resection at Tohoku University Hospital between January 2010 and December 2022. Histological responses were graded using the JPS system. Survival analyses were conducted using Kaplan–Meier estimation and Cox proportional hazards modeling.

**Results:**

Histological Grade 1a, 1b, 2, 3, and 4 responses were observed in 47 (40.2%), 42 (35.9%), 21 (17.9%), 6 (5.1%), and 1 (0.9%) patients, respectively. Patients with a Grade ≥ 2 response had significantly longer overall survival (OS) and significantly higher R0 resection rates than those with Grade 1a or 1b responses (median OS: 118.7 vs. 46.6 months, *p* = 0.037; 100% vs. 82.0%, *p* = 0.01). In a multivariable Cox regression model including RECIST progression status and baseline covariates, JPS Grade ≥ 2 remained significantly associated with better OS (adjusted HR 0.48; 95% CI 0.24–0.96; *p* = 0.039).

**Conclusion:**

Substantial histological response (JPS Grade ≥ 2) after NAC is strongly associated with improved OS and higher R0 resection rates in patients with resectable PDAC. The JPS grading system provides a basis for clinically meaningful prognostic stratification.

**Supplementary Information:**

The online version contains supplementary material available at https://doi.org/10.1007/s00595-026-03325-w.

## Introduction

Pancreatic ductal adenocarcinoma (PDAC) has the poorest prognosis among all malignancies. The persistently low 5-year survival rate of < 10% underscores the urgent need for more effective and innovative therapeutic strategies [1]. Currently, surgical resection is the only potential curative treatment for PDAC; however, because most patients present with advanced stage disease, curative surgery is feasible in only 15–20% of patients at diagnosis [2]. The high recurrence rates and suboptimal long-term outcomes highlight the importance of integrating systemic therapies, including chemotherapy and radiotherapy, into the treatment approach, particularly for patients who undergo resection [3, 4].

Neoadjuvant chemotherapy (NAC) has become a central component of the multidisciplinary treatment of pancreatic cancer [5, 6]. NAC enables early systemic control of micrometastatic disease, may reduce tumor size to enhance surgical resectability, and provides valuable insights into tumor biology [7]. Recently, the CISPD-1 phase 3 randomized controlled trial (NCT03750669) in patients with resectable pancreatic cancer demonstrated that sequential NAC with nab-paclitaxel plus gemcitabine followed by modified FOLFIRINOX significantly prolonged event-free survival (15.3 vs. 10.9 months; hazard ratio [HR], 0.71; *p* = 0.014) and overall survival (OS), increased R0 resection rates, and had a manageable safety profile [8].

In Japan, the randomized phase II/III Prep-02/JSAP05 trial recently validated the efficacy of NAC with gemcitabine plus S-1 (GS) compared with upfront surgery in patients with resectable PDAC [9, 10]. That study revealed a significant improvement in OS (median OS: 37.0 vs. 26.6 months; HR, 0.73; *p* = 0.018) and relapse-free survival (RFS) (median RFS: 14.3 vs. 11.3 months; HR, 0.77; *p* = 0.030) in patients who received neoadjuvant GS. Moreover, NAC was associated with significantly fewer postoperative liver metastases and lymph node-positivity, highlighting its role in systemic disease control and in reducing tumor burden before surgery. Despite this shift toward preoperative systemic therapy, data on the prognostic value of histological response assessment remain limited in Japanese clinical practice.

Several histological grading systems are available for assessing tumor regression following neoadjuvant therapy, each with distinct conceptual frameworks and criteria, making cross-system comparisons challenging [11]. Several studies have examined the relationship between histological treatment response and prognosis in pancreatic cancer; however, most have utilized classification systems developed in Western clinical settings, such as the College of American Pathologists (CAP) and Evans grading systems [12–15]. Few reports focus on the Japan Pancreas Society (JPS) classification, despite its widespread use in Japanese clinical practice. Moreover, most major studies have examined chemotherapy regimens developed in the West, and data specific to GS, the most commonly used neoadjuvant regimen in Japan, are scarce.

To address these gaps, we evaluated histological response using the JPS grading system in a large cohort of patients treated primarily with GS and examined its association with OS and curative resection. By validating the JPS system specifically, we aim to establish a more clinically relevant prognostic framework for use in Japanese clinical practice.

## Methods

### Study design and patients

The subjects of this retrospective study were 117 consecutive patients diagnosed with resectable pancreatic cancer who underwent NAC followed by curative-intent surgical resection at the Department of Surgery, Tohoku University Hospital, between January 2010 and December 2022.

Inclusion criteria were as follows: (1) age ≥ 20 years; (2) histologically confirmed invasive pancreatic ductal adenocarcinoma, including conventional PDAC and selected histological variants; (3) radiologically resectable disease based on National Comprehensive Cancer Network (NCCN) criteria [16]; and (4) treatment with NAC followed by surgical resection at the study institution. Tumor resectability was determined by a multidisciplinary team using contrast-enhanced computed tomography (CT) or magnetic resonance imaging (MRI), in accordance with NCCN guidelines. Exclusion criteria were as follows: (1) pancreatic tumors other than invasive pancreatic ductal adenocarcinoma and its selected histological variants, including acinar cell carcinoma, neuroendocrine carcinoma, or invasive carcinomas arising from intraductal papillary mucinous neoplasms or mucinous cystic neoplasms; (2) preoperative radiotherapy; and (3) incomplete clinical or pathological data.

This study was approved by the Institutional Review Board of Tohoku University (approval number: 2024-1-200) and was conducted in accordance with institutional and national ethical standards and the Declaration of Helsinki (1964) and its later amendments. The requirement for informed consent was waived because of the retrospective design.

### Neoadjuvant chemotherapy

Patients predominantly received NAC comprising gemcitabine (1000 mg/m² administered intravenously on days 1 and 8 every 3 weeks) combined with S-1 (80 mg/m²/day orally on days 1–14 every 3 weeks), or gemcitabine (1000 mg/m² administered weekly) combined with nab-paclitaxel (125 mg/m² administered weekly for 3 consecutive weeks every 4 weeks). Some patients received either gemcitabine monotherapy (1000 mg/m² administered intravenously on days 1, 8, and 15 every 4 weeks) or modified FOLFOX6, comprising oxaliplatin (85 mg/m²) and leucovorin (200 mg/m²) administered intravenously on day 1, followed by 5-fluorouracil (400 mg/m²) bolus and continuous infusion (2400 mg/m² over 46 h). Chemotherapy was administered for a median duration of two cycles.

### Surgical procedures

All patients in this study underwent curative-intent standard pancreatic resections, including pancreatoduodenectomy, distal pancreatectomy, total pancreatectomy, or remnant pancreatic resection, based on tumor location and clinical indications.

### Pathological assessment

Pathological evaluation of the resected specimens included histological classification and staging according to the Union for International Cancer Control (UICC) TNM classification system [17]. Histological treatment response was graded using the JPS grading system, which categorizes tumor response into four grades based on estimated residual tumor viability [18]. Grade 1 (poor or no response) was subdivided into Grade 1a (estimated residual cancer rate ≥ 90%) and Grade 1b (50% ≤ residual rate < 90%). Grade 2 indicated a moderate response (10% ≤ residual rate < 50%), Grade 3 indicated a marked response (residual rate < 10%), and Grade 4 indicated a complete response (no residual viable cancer cells). Histological treatment response was also assessed using the Evans grading system [12]: Grade I, minimal (< 10%) or no tumor cell destruction; Grade IIa, destruction of 10–50% of tumor cells; Grade IIb, destruction of 51–90% of tumor cells; Grade III, destruction of > 90% of tumor cells; and Grade IV, destruction of 100% of tumor cells.

Surgical margins were assessed carefully. In accordance with the JPS definition (0-mm rule), R0 resection was defined as the absence of tumor cells at the inked resection margin, indicating a microscopically negative margin. However, several Western studies apply a 1-mm clearance criterion, in which tumor cells within 1 mm of a margin are classified as R1; therefore, reported R0 rates may vary depending on the definition used. All pathological assessments were performed in accordance with routine institutional practice using the JPS and Evans criteria [12, 18]. Histological treatment response grading (JPS and Evans) and margin assessment were performed primarily by a board-certified pancreatobiliary pathologist. Where grading was ambiguous or discrepant on secondary review, the slides were re-evaluated jointly by two pathologists, and a final grade was assigned by consensus (multi-headed microscope review).

### Radiologic response assessment

Radiologic response to neoadjuvant chemotherapy was evaluated according to the Response Evaluation Criteria in Solid Tumors (RECIST) version 1.1. Baseline imaging obtained at diagnosis (before NAC) and post-NAC preoperative restaging imaging (contrast-enhanced CT or MRI) were reviewed. Response was categorized as complete response (CR), partial response (PR), stable disease (SD), or progressive disease (PD) based on the standard RECIST v1.1 definitions.

### Adjuvant chemotherapy

Postoperative adjuvant chemotherapy was administered according to standard clinical guidelines, typically starting within 10 weeks after surgery, depending on the patient’s recovery and performance status. Most patients received either S-1 monotherapy (80–120 mg/day orally, on a 4-weeks-on/2-weeks-off cycle) or gemcitabine-based regimens, including gemcitabine monotherapy (1000 mg/m² on days 1, 8, and 15 of a 28-day cycle). Generally, chemotherapy was continued for a planned duration of 6 months unless limited by toxicity or disease recurrence.

### Follow-up and survival analysis

OS and RFS were calculated from the date of surgery to the date of death and the date of first documented recurrence, respectively. Recurrence was defined as radiologic or pathologic evidence of disease relapse, confirmed by imaging studies or histological examination. Post-recurrence survival (PRS) was defined as the interval between the date of confirmed recurrence and the date of death or last follow-up. Follow-up was conducted from the date of surgery until death or the last confirmed survival status. Data collection was completed on May 31, 2025.

### Statistical analysis

Survival analyses were conducted using the Kaplan–Meier method, with intergroup comparisons assessed by the log-rank test. The prognostic impact of histological treatment response grades was evaluated using Cox proportional hazards regression models, with results reported as HRs and corresponding 95% confidence intervals (CIs). Continuous variables are summarized as medians with ranges.

To assess whether a substantial histological response (JPS Grade ≥ 2) was associated with OS and whether it provided prognostic information beyond conventional radiologic response, we performed a multivariable Cox proportional hazards regression model including JPS Grade ≥ 2, RECIST progression status (PD vs. non-PD), and clinically relevant baseline covariates (age, tumor location, pretreatment CA19-9 [log-transformed], pretreatment tumor size, and NAC regimen [GS vs. others]). Covariates were selected a priori to minimize overfitting, given the number of deaths. The incremental prognostic value of JPS Grade ≥ 2 beyond RECIST PD status was evaluated by comparing nested Cox models with and without JPS Grade ≥ 2, using the likelihood ratio test.

To identify factors independently associated with achieving a substantial histological response, defined as JPS Grade ≥ 2, multivariable logistic regression analysis was conducted. Odds ratios (ORs) and 95% CIs were calculated using the *statsmodels* package in Python. To minimize overfitting, given the limited number of events (JPS Grade ≥ 2, *n* = 28), we used a parsimonious multivariable logistic regression model, including a maximum of three clinically relevant preoperative covariates (tumor size on post-NAC preoperative imaging [post-NAC tumor size], tumor location, and preoperative CA19-9).

Baseline pretreatment tumor size was defined as the maximum diameter of the primary tumor, measured on baseline contrast-enhanced CT or MRI obtained at diagnosis before the initiation of NAC. Post-NAC tumor size was defined as the maximum diameter of the primary tumor measured on post-NAC preoperative restaging contrast-enhanced CT or MRI. The percentage change in tumor size from baseline was calculated as [(post-NAC tumor size - pretreatment tumor size) / pretreatment tumor size] × 100. Because surgical procedure was largely determined by tumor location, surgical procedure was not included in the multivariable model; instead, tumor location was included. All statistical analyses were conducted using GraphPad Prism version 10 (GraphPad Software, San Diego, CA) and Python 3.10. A two-tailed p-value < 0.05 was considered significant.

## Results

### Patient characteristics in the entire cohort

A total of 117 patients with resectable invasive PDAC underwent NAC followed by surgical resection at our institution between January 2010 and December 2022. Table 1 summarizes the patients’ characteristics. The cohort included 75 men (64.1%) and 42 women (35.9%), with a median age of 71 years (interquartile range [IQR]: 63–75 years; range: 41–89 years). In terms of age distribution, 28.2% were aged < 65 years, 39.3% were aged 65–74 years, and 32.5% were aged ≥ 75 years.

**Table 1:** Demographic, clinical, and pathologic characteristics of the entire cohort

Characteristics	All	Grade 1a+1b	Grade ≥2	*p*-value
	(*n* = 117)	(*n* = 89)	(*n* = 28)	
Age, year, median (IQR) — no. (%)	71 (63–75)	71 (63–75)	70 (64–77)	0.88
<65	33 (28.2)	26 (29.2)	7 (25.0)	
65–74	46 (39.3)	35 (39.3)	11 (39.3)	
≥75	38 (32.5)	28 (31.5)	10 (35.7)	
Sex — no. (%)				0.84
Male	75 (64.1)	58 (65.2)	17 (60.7)	
Female	42 (35.9)	31 (34.8)	11 (39.3)	
Pretreatment CA19-9, U/mL, median (IQR) — no. (%)	60.2 (15–208)	60.2 (13–287)	66.2 (16–150)	0.63
≤37 U/mL	48 (41.0)	35 (39.3)	13 (46.4)	
≤100 U/mL	18 (15.4)	13 (14.6)	5 (17.9)	
>100 U/mL	51 (43.6)	41 (46.1)	10 (35.7)	
Preoperative CA19-9, U/mL, median (IQR) — no. (%)	23.1 (11-74)	34.7 (11–78)	17.4 (12–59)	0.19
≤37 U/mL	62 (53.0)	45 (50.5)	17 (60.7)	
≤100 U/mL	33 (28.2)	24 (27.0)	9 (32.1)	
>100 U/mL	22 (18.8)	20 (22.5)	2 (7.2)	
Percent reduction in CA19-9, median	39.1	39.7	32.9	0.33
Neoadjuvant Chemotherapy (NAC) regimen — no. (%)				0.28
Gemcitabine plus S-1 (GS)	103 (88.0)	78 (87.7)	25 (89.3)	
Gemcitabine plus nab-paclitaxel (GnP)	12 (10.2)	10 (11.2)	2 (7.1)	
Gemcitabine	1 (0.9)	0	1 (3.6)	
mFOLFOX6	1 (0.9)	1 (1.1)	0	
Length of neoadjuvant period — no. (%)				1.00
≤60 days	103 (88.0)	78 (87.6)	25 (89.2)	
>60 days	14 (12.0)	11 (12.4)	3 (10.8)	
Pretreatment tumor size, mm, median (IQR) — no. (%)	21 (15–26)	21 (15–27)	20 (16–25)	0.45
≤20 mm	55 (47.0)	40 (45.0)	15 (53.6)	
21–40 mm	61 (52.1)	48 (53.9)	13 (46.4)	
>40 mm	1 (0.9)	1 (1.1)	0	
Post-NAC tumor size, mm, median (IQR) — no. (%)	21 (15–26)	22 (18–28)	15 (11–20)	**<0.01**
≤20 mm	55 (47.0)	34 (38.2)	21 (75.0)	
21–40 mm	59 (50.4)	52 (58.4)	7 (25.0)	
>40 mm	3 (2.6)	3 (3.4)	0	
Percentage change in tumor size from baseline, median (IQR) — no. (%)	0.0 (−20.0 to 15.8)	4.76 (−9.1 to 16.7)	−23.61 (−41.4 to −2.9)	**<0.01**
RECIST classification (version 1.1)				**<0.01**
Complete response (CR)	1 (0.9)	0	1 (3.6)	
Partial response (PR)	19 (16.1)	8 (9.0)	11 (39.2)	
Stable disease (SD)	85 (72.7)	70 (78.6)	15 (53.6)	
Progressive disease (PD)	12 (10.3)	11 (12.4)	1 (3.6)	
Tumor location — no. (%)				**<0.01**
Head	65 (55.6)	56 (62.9)	9 (32.1)	
Body/Tail	52 (44.4)	33 (37.1)	19 (67.9)	
Preoperative biliary drainage† — no. (%)				0.54
Yes	35 (53.8)	31 (55.4)	4 (44.4)	
No	30 (46.2)	25 (44.6)	5 (55.6)	
Drainage modality (primary)†				0.72
ERCP-based drainage — no. (%)	34 (97.1)	30 (96.8)	4 (100)	
PTBD only — no. (%)	1 (2.9)	1 (3.2)	0	
Stent type (if stented)† — no. (%)				0.22
Plastic stent	31 (91.2)	28 (93.3)	3 (75.0)	
Self-expandable metal stent (SEMS)	3 (8.8)	2 (6.7)	1 (25.0)	
Biliary infection before surgery† — no. (%)				0.12
Yes	12 (18.5)	12 (21.4)	0	
No	53 (81.5)	44 (78.6)	9 (100)	
Surgical procedure — no. (%)				**<0.01**
Pancreatoduodenectomy (PD)	59 (50.4)	50 (56.2)	9 (32.1)	
Distal pancreatectomy (DP)	45 (38.5)	27 (30.3)	18 (64.3)	
Total pancreatectomy (TP)	13 (11.1)	12 (13.5)	1 (3.6)	
Operation time, median (range), min	488 (197-720)	500 (245-720)	455 (197-704)	0.09
Blood loss, median (range), mL	814 (34-4385)	871 (34-4385)	519 (63-1989)	**0.02**
Complications (Clavien-Dindo classification grade) — no. (%)				0.96
0–Ⅱ	86 (73.5)	67 (75.3)	19 (67.9)	
Ⅲ	28 (23.9)	20 (22.5)	8 (28.5)	
Ⅳ	3 (2.6)	2 (2.2)	1 (3.6)	
Ⅴ	0	0	0	
Postoperative hospital stays, median (range), days	23 (9-110)	23 (10-110)	23 (9-54)	0.27
Intraperitoneal washing cytology — no. (%)				
Negative	117 (100)	89 (100)	28 (100)	
Positive	0	0	0	
Histological grade — no. (%)				**0.01**
Well	37 (31.7)	33 (37.1)	4 (14.3)	
Mod	61 (52.1)	41 (46.1)	20 (71.5)	
Por	6 (5.1)	4 (4.5)	2 (7.1)	
Adenosqua	6 (5.1)	6 (6.7)	0	
Anaplastic	4 (3.4)	4 (4.5)	0	
Muc	1 (0.9)	1 (1.1)	0	
Not specified	2 (1.7)	0	2 (7.1)	
Tumor grade — no. (%)				**0.02**
G1	37 (31.6)	33 (37.1)	4 (14.3)	
G2	61 (52.1)	42 (47.1)	19 (67.9)	
G3	12 (10.3)	7 (7.9)	5 (17.8)	
G4	7 (6.0)	7 (7.9)	0	
UICC 8th T category — no. (%)				**0.01**
T0	1 (0.9)	0	1 (3.6)	
T1	40 (34.2)	25 (28.1)	15 (53.6)	
T2	33 (28.2)	30 (33.7)	3 (10.7)	
T3	43 (36.7)	34 (38.2)	9 (32.1)	
UICC 8th N category — no. (%)				**0.03**
N0	50 (42.7)	37 (41.6)	13 (46.4)	
N1	43 (36.8)	29 (32.6)	14 (50.0)	
N2	24 (20.5)	23 (25.8)	1 (3.6)	
UICC 8th Stage — no. (%)				**<0.01**
Stage 0	1 (0.9)	0	1 (3.6)	
Stage IA	23 (19.7)	13 (14.6)	10 (35.7)	
Stage IB	10 (8.5)	10 (11.2)	0	
Stage IIA	17 (14.5)	14 (15.7)	3 (10.7)	
Stage IIB	42 (35.9)	28 (31.5)	14 (50.0)	
Stage III	20 (17.1)	20 (22.5)	0	
Stage Ⅳ	4 (3.4)	4 (4.5)	0	
Lymphatic invasion — no. (%)				**<0.01**
Negative	32 (27.3)	18 (20.2)	14 (50.0)	
Positive	85 (72.7)	71 (79.8)	14 (50.0)	
Venous invasion — no. (%)				0.24
Negative	4 (3.4)	2 (2.2)	2 (7.1)	
Positive	113 (96.6)	87 (97.8)	26 (92.9)	
Perineural/neural invasion — no. (%)				0.12
Negative	17 (14.5)	10 (11.2)	7 (25.0)	
Positive	100 (85.5)	79 (88.8)	21 (75.0)	
Margin status — no. (%)				**0.01**
R0	101 (86.3)	73 (82.0)	28 (100)	
R1	16 (13.7)	16 (18.0)	0	
Histological treatment response (JPS) — no. (%)				-
Grade 1a	47 (40.2)	47 (52.9)	-	
Grade 1b	42 (35.9)	42 (47.1)	-	
Grade 2	21 (17.9)	-	21 (75.0)	
Grade 3	6 (5.1)	-	6 (21.4)	
Grade 4	1 (0.9)	-	1 (3.6)	
Histological treatment response (Evans) — no. (%)				-
Grade I	43 (36.8)	42 (47.1)	1 (3.6)	
Grade IIa	52 (44.3)	43 (48.4)	9 (32.1)	
Grade IIb	18 (15.4)	4 (4.5)	14 (50.0)	
Grade III	3 (2.6)	0	3 (10.7)	
Grade Ⅳ	1 (0.9)	0	1 (3.6)	
Receipt of adjuvant therapy — no. (%)	107 (91.5)	79 (88.8)	28 (100)	0.12

† Percentages calculated among pancreatic head tumors only

Abbreviations: ERCP, endoscopic retrograde cholangiopancreatography; PTBD, percutaneous transhepatic biliary drainage

RECIST, Response Evaluation Criteria in Solid Tumors

Bold values represent significant p-values

GS was the predominant NAC regimen, administered to 103 (88.0%) patients, followed by gemcitabine plus nab-paclitaxel (GnP) in 12 (10.2%) patients. Gemcitabine monotherapy and modified FOLFOX6 were each used in one patient. The median pretreatment carbohydrate antigen 19 − 9 (CA19-9) level was 60.2 U/mL (IQR: 15–208 U/mL), which decreased to a median of 23.1 U/mL (IQR: 11–74 U/mL) before surgery, indicating a substantial reduction following NAC. Most patients (88.0%) completed neoadjuvant treatment in fewer than 60 days.

### Surgical and pathological findings of the entire cohort

The surgical procedures performed were pancreatoduodenectomy (PD) in 59 patients (50.4%), distal pancreatectomy (DP) in 45 (38.5%), and total pancreatectomy (TP) (including residual pancreatectomy) in 13 (11.1%). The median operation time was 488 min (range: 197–720 min), and the median intraoperative blood loss was 814 mL (range: 34–4385 mL). Postoperative complications, classified according to the Clavien–Dindo system, were Grade 0–II in 86 patients (73.5%), Grade III in 28 (23.9%), and Grade IV in 3 (2.6%). There were no Grade V complications. The median postoperative hospital stay was 23 days (range: 9–110 days).

Histopathological analysis identified conventional PDAC in 104 (88.9%) patients, with histological variants including adenosquamous carcinoma in six patients (5.1%), anaplastic carcinoma in four patients (3.4%), and mucinous carcinoma in one patient (0.9%). The histological subtype was not specified in two patients (1.7%). According to the UICC eighth edition pathological staging, one (0.9%) patient had stage 0, 33 (28.2%) had stage I, 59 (50.4%) had stage II, 20 (17.1%) had stage III, and 4 (3.4%) had stage IV disease. The R0 resection rate was 86.3%. The histological response to NAC, assessed using the standardized JPS criteria, was distributed as follows: Grade 1a in 47 (40.2%) patients, Grade 1b in 42 (35.9%), Grade 2 in 21 (17.9%), Grade 3 in six (5.1%), and Grade 4 in one (0.9%). Using the Evans grading system, responses were classified as Grade I in 43 (36.8%) patients, Grade IIa in 52 (44.3%), Grade IIb in 18 (15.4%), Grade III in 3 (2.6%), and Grade IV in 1 (0.9%). Adjuvant chemotherapy was initiated in 91.5% of the patients.

### Histological response and OS of the entire cohort

**Fig. 1:**
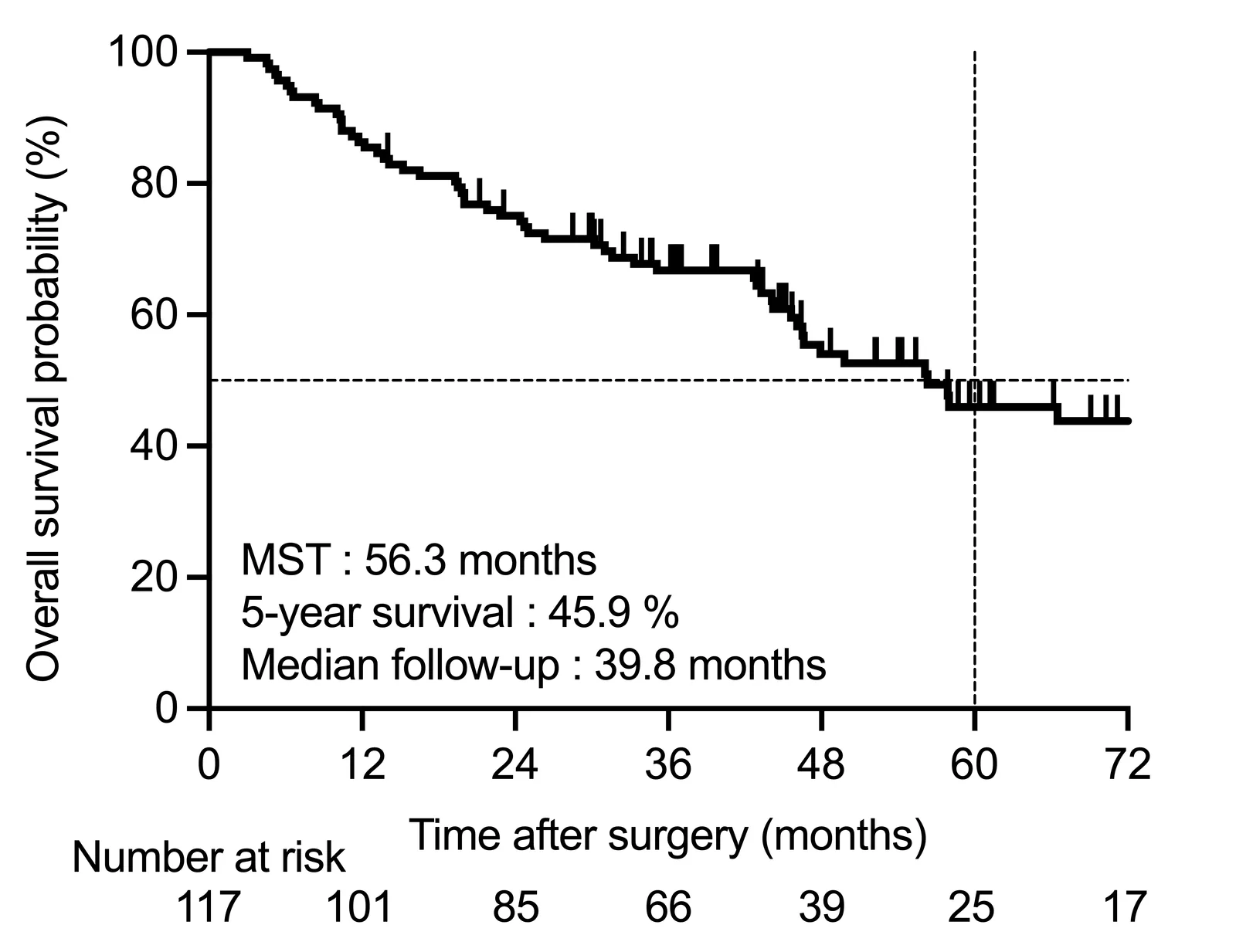
Overall survival of the entire cohort. Kaplan–Meier estimates of overall survival are shown for the entire cohort. The median survival time was 56.3 months, with a 5-year overall survival rate of 45.9%. The median follow-up duration was 39.8 months

**Fig. 2:**
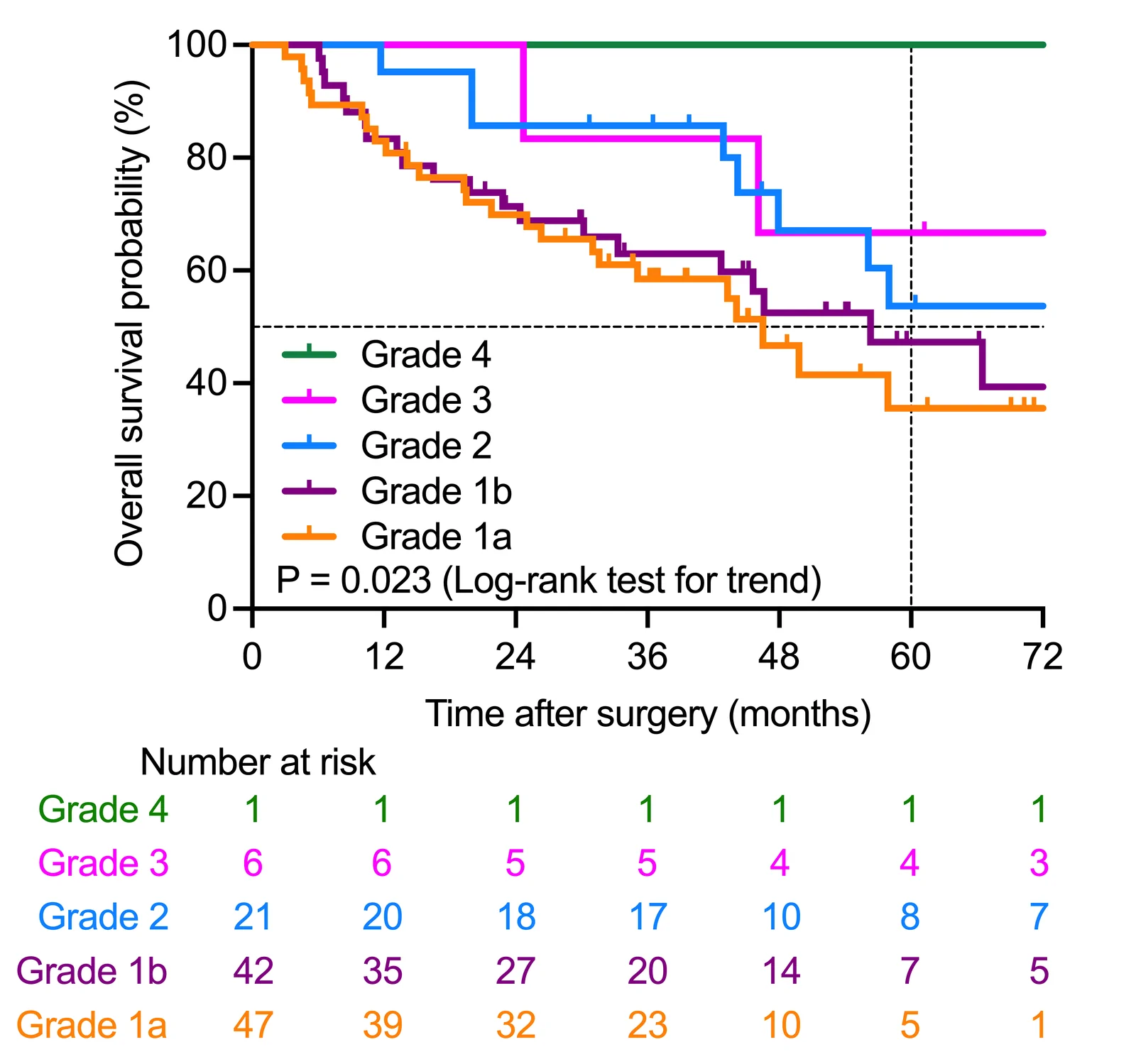
Overall survival stratified by the JPS histological treatment response grade. Kaplan–Meier curves of overall survival based on histological response grades, using the Japan Pancreas Society (JPS) grading system: Grade 4 (green), 3 (magenta), 2 (light blue), 1b (purple), and 1a (orange). A stepwise decline in survival was observed with decreasing histological response (log-rank test for trend, *p* = 0.023)

Figure 1 illustrates the Kaplan–Meier curves for OS in the cohort that received NAC followed by surgical resection. The median survival time was 56.3 months, with a 5-year OS rate of 45.9% and a 39.8-month median follow-up duration. Stratification by histological treatment response, according to the JPS grading system, revealed a significant survival trend across response grades. Patients were classified into five groups (Grades 1a, 1b, 2, 3, and 4), and Kaplan–Meier analysis demonstrated a significant association between histological response grade and OS (log-rank test for trend, *p* = 0.023) (Fig. 2). In an exploratory comparison using the Evans histological treatment response grading system, patients were stratified similarly into five groups (Grades I, IIa, IIb, III, and IV); however, the OS trend across Evans grades was not significant (log-rank test for trend, *p* = 0.17) (Online Resource 1). Collectively, these results suggest that a stronger histological response, indicated by higher JPS grades, is associated with improved long-term survival, underscoring the prognostic utility of the JPS grading system in the neoadjuvant treatment setting for pancreatic cancer.

### Clinicopathological subgroup analysis for baseline and preoperative characteristics using the JPS histological treatment response grade

Subgroup analysis based on histological treatment response identified 89 patients (76.1%) with a low response (Grade 1a or 1b, collectively referred to as Grade 1a + 1b) and 28 patients (23.9%) with a high response (Grade ≥ 2) (Table 1). There were no significant differences between the two groups in age distribution (*p* = 0.88) or sex (*p* = 0.84). Pretreatment serum CA19-9 levels were also comparable between the low- and high-response groups (median: 60.2 U/mL [IQR, 13–287] vs. 66.2 U/mL [IQR, 16–150], *p* = 0.63). The median preoperative CA19-9 level was lower in the high-response group than in the low-response group (median: 17.4 U/mL [IQR, 12–59] vs. 34.7 U/mL [IQR, 11–78], *p* = 0.19). The median percent reduction in CA19-9 during NAC did not differ significantly between the groups (39.7% vs. 32.9%, *p* = 0.33). The GS regimen was administered to 87.7% and 89.3% of patients in the Grade 1a + 1b and Grade ≥ 2 groups, respectively, while GnP was administered to 11.2% and 7.1%, respectively (*p* = 0.28). The duration of neoadjuvant therapy did not differ significantly between the cohorts (*p* = 1.00). Pretreatment tumor size measured on baseline imaging was also comparable between the low- and high-response groups (median [IQR], 21 [15–27] mm vs. 20 [16–25] mm; *p* = 0.45), and the distribution across size categories was similar. Overall, the two groups were broadly comparable at baseline and received similar neoadjuvant treatment.

Significant differences were observed between the low- (Grade 1a + 1b) and high-response (Grade ≥ 2) groups across several clinicopathological parameters. The tumor location differed significantly between the groups (*p* < 0.01), with tumors in the pancreatic body or tail being more common in the Grade ≥ 2 group (67.9%) than in the Grade 1a + 1b group (37.1%). In contrast, tumors in the pancreatic head were more frequently observed in the Grade 1a + 1b group (62.9% vs. 32.1%).

In the analyses restricted to tumors located in the pancreatic head, preoperative biliary management was broadly comparable between the Grade 1a + 1b and Grade ≥ 2 groups. The rate of preoperative biliary drainage did not differ significantly between these groups (55.4% vs. 44.4%, *p* = 0.54). Among the patients who underwent drainage, endoscopic retrograde cholangiopancreatography predominated in both groups (96.8% vs. 100%), with percutaneous transhepatic biliary drainage alone being rare (3.2% vs. 0%, *p* = 0.72). Stent type distribution did not differ significantly between the groups (plastic stent: 93.3% vs. 75.0%; SEMS: 6.7% vs. 25.0%, *p* = 0.22). Preoperative biliary infection developed in 21.4% of the Grade 1a + 1b group patients and 0% of the Grade ≥ 2 group patients (*p* = 0.12). Overall, these results suggest that differences in histological response are unlikely to be explained by variability in preoperative biliary drainage practice within pancreatic head tumors.

### Clinicopathological subgroup analysis for perioperative and pathological findings using the JPS histological treatment response grade

The type of surgical procedure varied significantly between the groups (*p* < 0.01). DP was performed more frequently in the Grade ≥ 2 group (64.3%) than in the Grade 1a + 1b group (30.3%), whereas PD was more prevalent in the Grade 1a + 1b group (56.2% vs. 32.1%). Intraoperative blood loss was significantly lower in the high-response group, with a median loss of 519 mL (range: 63–1989 mL) vs. 871 mL (range: 34–4385 mL) in the low-response group (*p* = 0.02).

Histological differentiation differed significantly between the groups (*p* = 0.01). Tumor grade, based on the WHO classification, also differed significantly (*p* = 0.02), with Grade 2 (G2) tumors being more common in the Grade ≥ 2 group (67.9%) and G1 tumors being more common in the Grade 1a + 1b group (37.1%). The post-NAC tumor size was significantly lower in the Grade ≥ 2 group (median: 15 mm [IQR: 11–20 mm]) than in the Grade 1a + 1b group (22 mm [IQR: 18–28 mm], *p* < 0.01). Consistently, T-category distribution based on the UICC 8th edition differed between the groups (*p* = 0.01), with a higher proportion of T1 tumors in the high-response group (53.6% vs. 28.1%) and fewer T2–T3 tumors. The percentage change in tumor size from baseline was significantly greater in patients with Grade ≥ 2 response than in those with Grade 1a + 1b response (median [IQR], − 23.61% [− 41.4 to − 2.9] vs. +4.76% [− 9.1 to 16.7], *p* < 0.01).

Radiologic response to NAC was assessed using RECIST version 1.1 on post-NAC preoperative restaging imaging. The RECIST responses in the overall cohort were CR in 1 (0.9%) patient, PR in 19 (16.1%), SD in 85 (72.7%), and PD in 12 (10.3%) (Table 1). An objective radiologic response (CR/PR) was significantly more frequent in patients with a substantial histological response (Grade ≥ 2) than in those with Grade 1a + 1b disease (42.9% vs. 9.0%; Fisher’s exact *p* = 0.00014; OR 7.59 [95% CI, 2.68–21.55]). RECIST PD was associated with worse OS than non-PD (log-rank *p* = 0.044) (Online Resource 2).

Pathological staging revealed advanced disease (stage III–IV) in 27.0% of the patients with a Grade 1a + 1b response but in 0% of those with a Grade ≥ 2 response (*p* < 0.01), suggesting a more favorable pathological profile in patients with higher histological grades. Lymphatic invasion was observed in both groups but was significantly more prevalent in the Grade 1a + 1b group (*p* < 0.01). Venous invasion was nearly universal in both groups. Perineural invasion was slightly less common in the Grade ≥ 2 group (75.0% vs. 88.8%), although the difference was not significant. Notably, the R0 resection rate was significantly higher in the Grade ≥ 2 group (100%) than in the Grade 1a + 1b group (82.0%, *p* = 0.01). Furthermore, a higher proportion of Grade ≥ 2 responders received adjuvant therapy (100% vs. 88.8%), but again, the difference was not significant (*p* = 0.12).

Collectively, these findings underscore that a higher histological treatment response (Grade ≥ 2) was associated with more favorable pathological features, including smaller tumor size, reduced lymphatic invasion, and higher R0 resection rates.

### Comparison of OS between the JPS histological response grade low-response (Grade 1a + 1b) and high-response (Grade ≥ 2) Groups

**Fig. 3:**
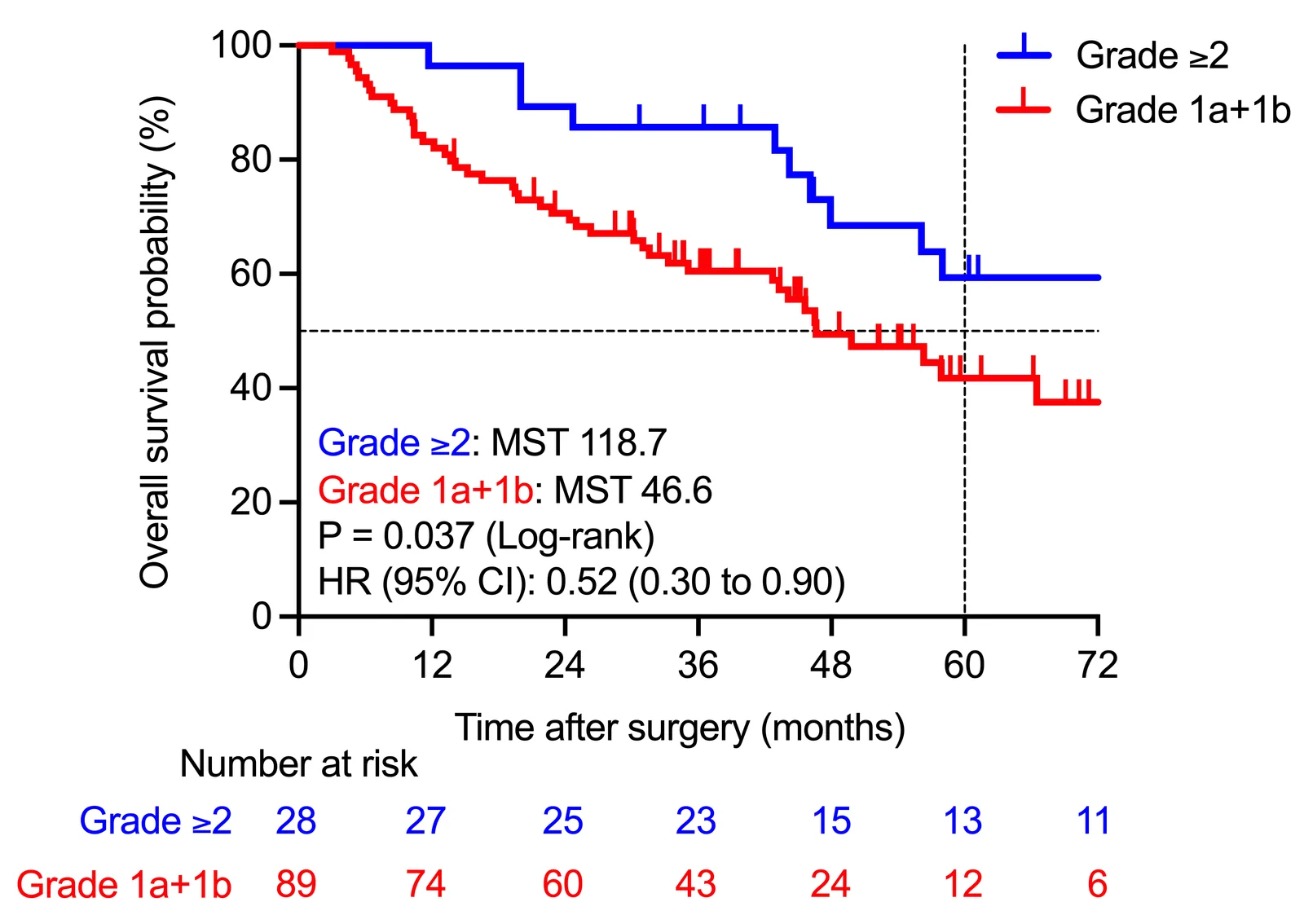
Overall survival of patients with low (JPS Grade 1a + 1b) vs. high (JPS Grade ≥ 2) histological response. Kaplan–Meier analysis of overall survival comparing patients with low (Grade 1a + 1b; red) and high (Grade ≥ 2; blue) histological responses following neoadjuvant chemotherapy using the Japan Pancreas Society (JPS) grading system. Overall survival was significantly better in the Grade ≥ 2 group (log-rank test, *p* = 0.037)

There was no significant difference in OS between patients with Grade 1a and Grade 1b responses (median OS: 46.5 vs. 56.3 months; HR, 0.82; 95% CI: 0.46–1.45; log-rank *p* = 0.49), indicating comparable long-term outcomes between these two low-response subgroups (Fig. 2). Conversely, patients with a higher treatment response (Grade ≥ 2) had significantly longer OS than those with Grade 1a or 1b responses (combined Grade 1a + 1b group), with median OS of 118.7 and 46.6 months, respectively (HR, 0.52; 95% CI: 0.30–0.90; log-rank *p* = 0.037; Fig. 3). For comparison, OS was also evaluated using the Evans histological treatment response grading system by dichotomizing patients into low responders (Grade I + IIa) and high responders (Grade ≥ IIb). Although the high-response group had longer OS than the low-response group did, the difference was not significant (median: 72.2 vs. 47.9 months; HR, 0.58; 95% CI: 0.32–1.06; log-rank *p* = 0.13) (Online Resource 3). Taken together, these results suggest that JPS-based response categories provide more prognostic separation in this cohort, supporting their clinical relevance for post-neoadjuvant treatment risk assessment and outcome prediction.

In a multivariable Cox regression model adjusting for baseline covariates (age, tumor location, pretreatment CA19-9 [log-transformed], pretreatment tumor size, and NAC regimen [GS vs. others]) and RECIST progression status (PD vs. non-PD), JPS Grade ≥ 2 remained significantly associated with better OS (adjusted HR 0.48; 95% CI 0.24–0.96; *p* = 0.039). RECIST PD was significantly associated with worse OS (adjusted HR 2.89; 95% CI 1.29–6.45; *p* = 0.010) (Table 2). Adding JPS Grade ≥ 2 to the RECIST-based model improved model fit significantly (likelihood ratio test *p* = 0.030).

**Table 2:** Multivariable Cox proportional hazards regression analysis for overall survival including RECIST progression status (PD vs. non-PD)

Variable	adjusted HR	95% Confidence Interval	*p*-value
JPS Grade ≥2 (vs. Grade 1a+1b)	0.48	0.24–0.96	**0.039**
RECIST: PD (vs. non-PD)	2.89	1.29–6.45	**0.010**
Age (per 1-year increase)	1.06	1.02–1.09	**0.003**
Tumor location: Head (vs. Body/Tail)	0.85	0.48–1.51	0.579
Pretreatment CA19-9 (log-transformed)	1.06	0.91–1.24	0.423
Pretreatment tumor size (per 1-mm increase)	1.01	0.97–1.05	0.668
NAC regimen: GS (vs. others)	0.74	0.37–1.51	0.414

HR, Hazard Ratio; JPS, Japan Pancreas Society

RECIST, Response Evaluation Criteria in Solid Tumors; PD, Progressive disease

NAC, Neoadjuvant Chemotherapy; GS, Gemcitabine plus S-1

Bold values represent significant p-values

**Fig. 4:**
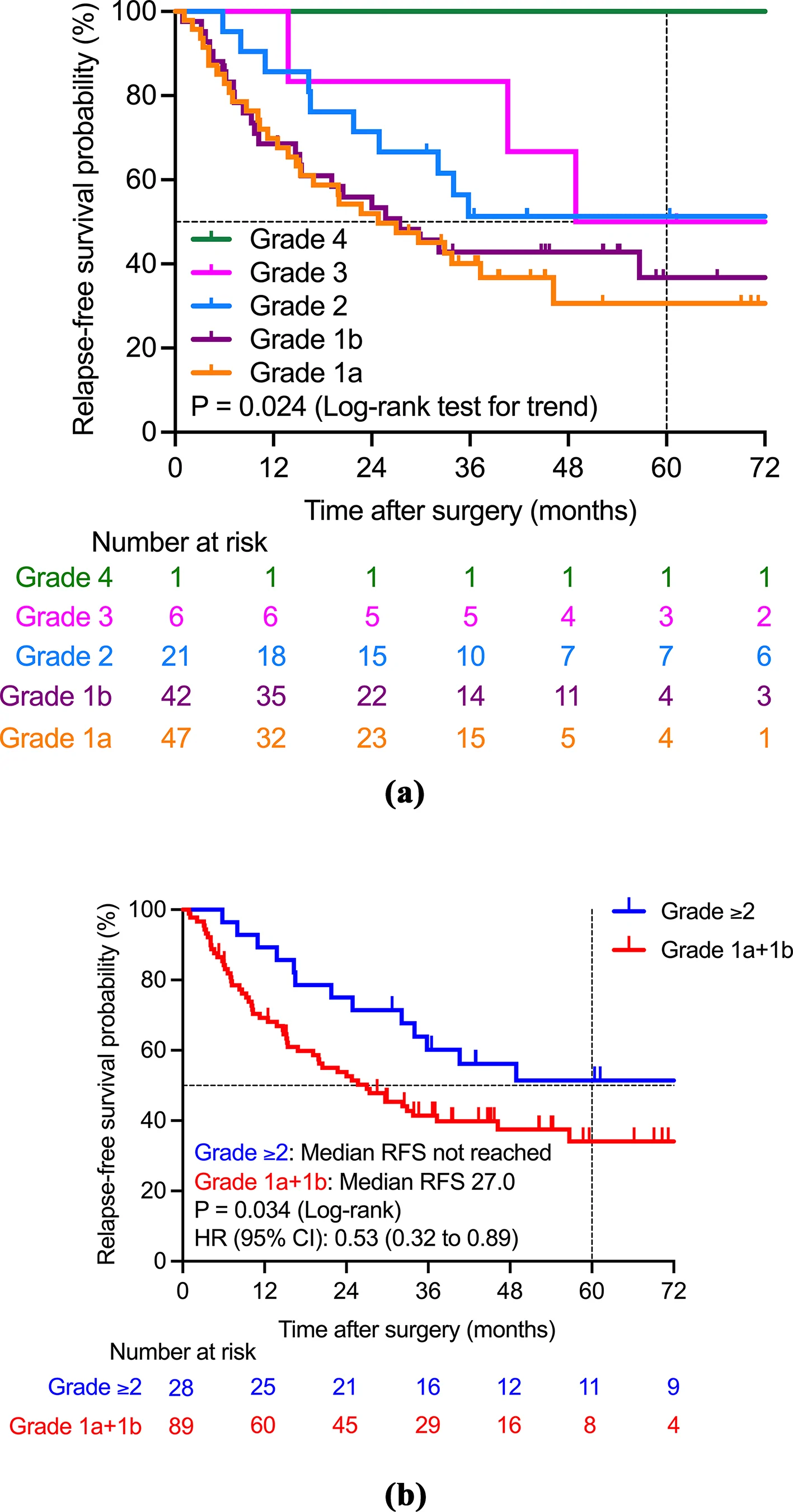
**a.** Relapse-free survival stratified by the JPS histological treatment response grade. Kaplan–Meier curves of relapse-free survival based on the Japan Pancreas Society (JPS) histological grades: Grade 4 (green), 3 (magenta), 2 (light blue), 1b (purple), and 1a (orange). A stepwise decrease in relapse-free survival was observed with lower histological response grades (log-rank test for trend, *p* = 0.024). **b**. Relapse-free survival in patients with low (JPS Grade 1a + 1b) vs. high (JPS Grade ≥ 2) histological response. Kaplan–Meier curves comparing relapse-free survival between patients with Grade 1a + 1b (red) and Grade ≥ 2 (blue) histological responses, using the Japan Pancreas Society (JPS) grading system. Relapse-free survival was significantly shorter in the Grade 1a + 1b group (log-rank test, *p* = 0.034)

**Fig. 5:**
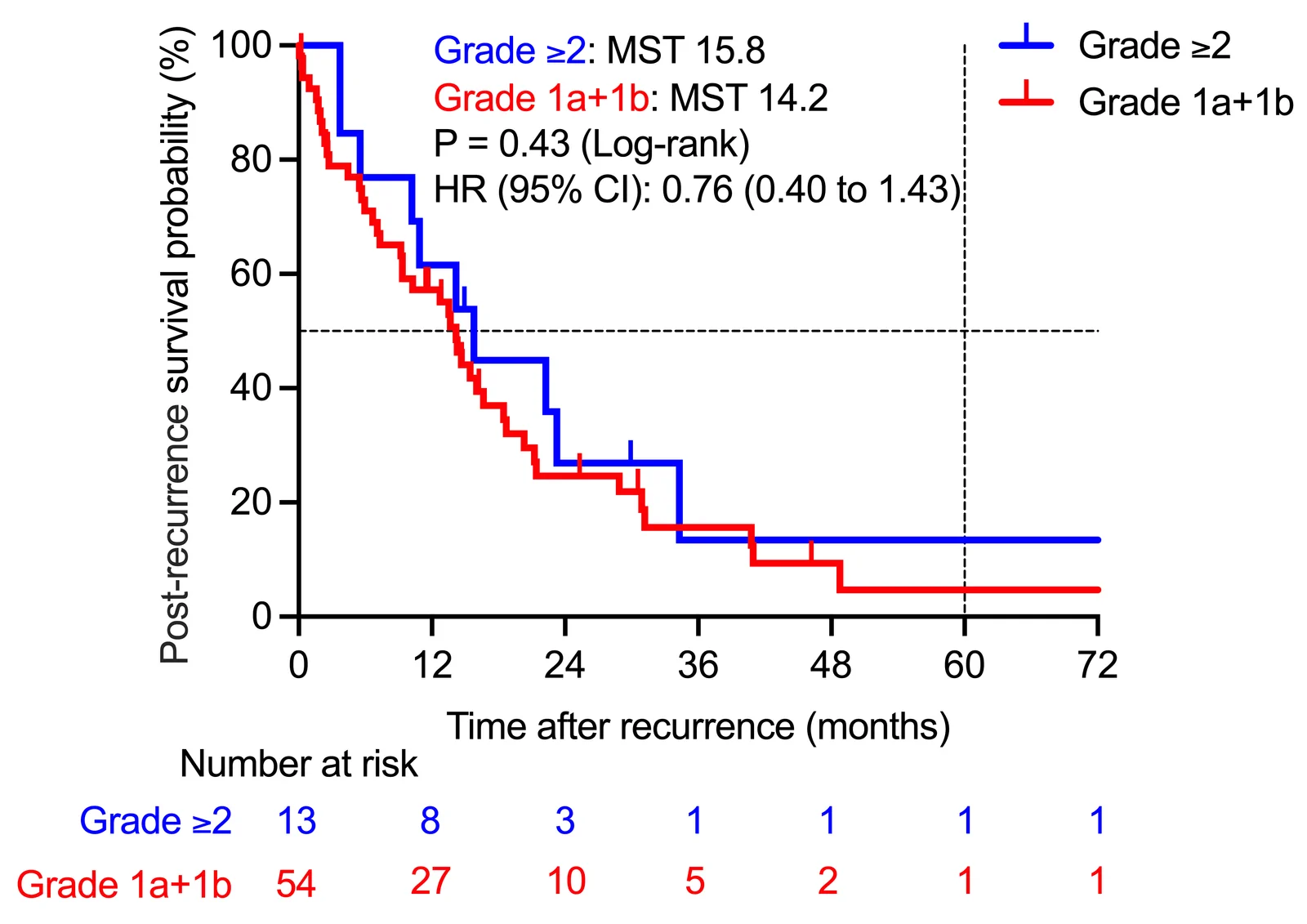
Kaplan–Meier curves of post-recurrence survival comparing patients with Grade 1a + 1b (red) and Grade ≥ 2 (blue) histological responses using the Japan Pancreas Society (JPS) grading system. No significant difference in post-recurrence survival was observed between the two groups (log-rank test, *p* = 0.43)

The JPS grading system classification revealed that RFS was significantly associated with the histological response grade (log-rank test for trend, *p* = 0.024) (Fig. 4a). Similar to OS, RFS was significantly better for patients with a Grade ≥ 2 response than for those with a Grade 1a + 1b response (median RFS: not reached vs. 27.0 months; HR, 0.53; 95% CI: 0.32–0.89; log-rank *p* = 0.034) (Fig. 4b). This underscores the prognostic value of achieving a robust histological response. However, there was no significant difference in PRS between the two cohorts (median PRS: 15.8 vs. 14.2 months; HR, 0.76; 95% CI: 0.40–1.43; log-rank *p* = 0.43) (Fig. 5), suggesting that the degree of initial pathological response may not influence survival outcomes following recurrence. Furthermore, the chemotherapy rate following recurrence did not differ significantly between the groups, although detailed recurrence patterns (such as local vs. distant and organ-specific sites) and post-recurrence treatment strategies were not systematically available in this retrospective dataset. Accordingly, the results of the PRS analysis should be interpreted with caution.

### Multivariable analysis of predictors in the high-response (Grade ≥ 2) group

In the parsimonious multivariable logistic regression model limited to three preoperative covariates (post-NAC tumor size, tumor location, and preoperative CA19-9 level), larger post-NAC tumor size was independently associated with lower odds of achieving a JPS Grade ≥ 2 response (OR per 1-mm increase, 0.88; 95% CI, 0.82–0.95; *p* = 0.001). Patients with tumors located in the pancreatic head were less likely to have a Grade ≥ 2 response than those with tumors located in the body or tail (OR for head vs. body/tail, 0.29; 95% CI, 0.11–0.77; *p* = 0.013). Preoperative CA19-9 levels (log-transformed) were not significantly associated with a Grade ≥ 2 response (OR, 1.11; 95% CI, 0.79–1.56; *p* = 0.538) (Table 3). Overall, these results suggest that both residual tumor burden on post-NAC imaging and the anatomical site affect histological response, with smaller tumors and a body or tail location more likely to result in a Grade ≥ 2 response. This supports a location-aware interpretation of treatment response after NAC.

**Table 3:** Multivariable logistic regression analysis of factors associated with substantial histological response (JPS Grade ≥ 2)

Variable	Odds Ratio (OR)	95% Confidence Interval	*p*-value
Post-NAC tumor size (per 1-mm increase)	0.88	0.82–0.95	**0.001**
Tumor location (head vs. body/tail)	0.29	0.11–0.77	**0.013**
Preoperative CA19-9 (log-transformed)	1.11	0.79–1.56	0.538

JPS, Japan Pancreas Society; NAC, Neoadjuvant Chemotherapy

Bold values represent significant p-values

## Discussion

This study comprehensively evaluated the prognostic relevance of histological treatment response, graded using the JPS system, in patients with resectable PDAC treated with NAC. The key findings were that a substantial histological response (Grade ≥ 2) was associated with significantly prolonged OS and that this response predicted a higher likelihood of R0 resection. These results highlight the potential of the JPS grading system as a retrospective evaluative tool and a practical prognostic biomarker to support individualized postsurgical management strategies. Although RECIST-based radiologic response remains the standard approach for monitoring NAC, objective responses were observed in a minority of patients, and most were classified as stable disease. In this context, JPS Grade ≥ 2 was associated with OS even after adjustment for RECIST progression status (PD vs. non-PD), and it improved model fit significantly, suggesting additional prognostic value beyond conventional size-based imaging response assessment.

Currently, several histological grading systems are used to assess treatment response following NAC for PDAC. The CAP system, used widely in Western clinical settings, evaluates absolute residual tumor volume without accounting for pretreatment tumor burden [13, 19]. The Evans grading system measures the extent of tumor cell destruction but lacks a standardized interpretation threshold [12]. In contrast, the JPS system considers both residual cancer and treatment-induced histological changes, such as necrosis and fibrosis [18]. However, comparative studies among these systems are limited and no international consensus has been established [20]. While numerous reports have demonstrated a correlation between higher histological response grades and improved survival of PDAC patients [14, 21–24], the most prognostically meaningful cutoff point remains inconsistent across studies.

In this context, recent consensus statements have advocated a conceptual shift in the post-neoadjuvant treatment pathological assessment of PDAC toward quantifying the viable residual tumor component rather than interpreting tumor regression using morphology. The 2019 Amsterdam International Consensus Meeting and the Neoadjuvant Therapy Working Group of the Pancreatobiliary Pathology Society emphasized that regression-related changes such as fibrosis, necrosis, and inflammation can be difficult to interpret in PDAC, given its intrinsically desmoplastic stroma and variability in sampling, and may contribute to the inconsistent prognostic performance reported for traditional, non–viability-centered tumor response grading systems such as CAP, the MD Anderson system, and the Evans grading system [12, 14, 15]. Adopting a viability-centered framework, supported by standardized specimen handling and sampling, may be essential for more objective and reproducible comparisons of NAC regimens and for improving prognostic accuracy [11, 25].

In line with this concept of assessing response by the residual viable tumor, the present study demonstrates that histological treatment response graded by the JPS system provides clinically meaningful prognostic stratification after NAC for resectable PDAC. There was no significant difference in OS between patients with Grade 1a and those with Grade 1b responses, although patients with a more substantial histological response (Grade ≥ 2) had significantly better OS and RFS. Importantly, this association with OS remained significant in a multivariable Cox regression model adjusting for RECIST progression status (PD vs. non-PD) and key baseline covariates, including NAC regimen (GS vs. others). These findings suggest that categorizing patients into low (Grade 1a + 1b) and high (Grade ≥ 2) responders offers meaningful prognostic stratification, aligning with the underlying biological rationale that greater tumor regression indicates enhanced chemosensitivity and a reduced residual disease burden. Notably, patients achieving JPS Grade ≥ 2 also exhibited several favorable clinicopathological features, including a smaller tumor size on post-NAC preoperative imaging, a higher proportion of body and tail tumors, earlier pathological stage, and higher R0 resection rates. While some of these factors may reflect intrinsic tumor biology and baseline disease burden, other factors, particularly yp stage and margin status, may also represent the downstream consequences of treatment effects. Therefore, JPS Grade ≥ 2 is best interpreted as an integrative on-treatment surrogate marker that captures residual tumor burden and treatment sensitivity (and, by extension, favorable tumor biology), rather than as a prognostic factor fully independent of all clinicopathological characteristics.

These findings are especially relevant to the Japanese clinical setting, where GS-based NAC is commonly used. The predominance of GS regimens in our cohort enhances the real-world applicability and external validity of the JPS grading system. Moreover, identifying Grade ≥ 2 as a favorable threshold may provide a rational basis for tailoring adjuvant therapy intensity according to treatment response.

Interestingly, PRS did not differ significantly between the groups, suggesting that the degree of initial histological response may have limited influence on disease progression or response to subsequent therapy after recurrence. This aligns with the findings of previous reports suggesting that post-recurrence outcomes are more strongly influenced by the biology of recurrent disease and the effectiveness of salvage treatments than by the initial treatment response. These findings highlight the need for improved therapeutic strategies and surveillance following recurrence, even in patients who respond well initially to NAC.

Furthermore, our findings emphasize that marked pathological regression significantly enhances the likelihood of R0 resection. Notably, a 100% R0 resection rate was achieved in patients with a Grade ≥ 2 response, highlighting the role of effective NAC in reducing tumor volume and enhancing the feasibility of complete surgical removal. Because margin status was defined using the JPS 0-mm rule (tumor not at ink), comparisons of R0 rates with those of studies adopting a 1-mm clearance criterion should be interpreted with caution. These results underscore the importance of integrating detailed histopathological assessments into routine clinical practice to accurately identify patients most likely to benefit from surgical interventions following chemotherapy.

In our cohort of resectable PDAC treated with NAC, smaller post-NAC tumor size was independently associated with a substantial histological response (JPS Grade ≥ 2). Notably, pretreatment tumor size on baseline imaging was comparable between the Grade 1a + 1b and Grade ≥ 2 groups (median [IQR], 21 [15–27] mm vs. 20 [16–25] mm; *p* = 0.45), whereas the percentage change in tumor size from baseline differed markedly (+ 4.76% [− 9.1 to 16.7] vs. −23.61% [− 41.4 to − 2.9]; *p* < 0.01). This supports the interpretation that smaller post-NAC tumor size primarily reflects treatment-associated tumor shrinkage and reduced residual tumor burden rather than earlier-stage disease at baseline.

A Grade ≥ 2 response was less likely in patients with tumors located in the pancreatic head than in those with tumors located in the body/tail. Post-NAC tumor size likely reflects residual tumor burden after treatment rather than baseline tumor burden. Thus, the association between smaller post-NAC preoperative imaging tumor size and achieving JPS Grade ≥ 2 is biologically plausible, as a smaller lesion on restaging imaging is more likely to reflect a lower residual tumor burden rather than merely an earlier baseline stage. Accordingly, post-NAC tumor size should be interpreted as an on-treatment marker of treatment efficacy and chemosensitivity rather than as a purely pretreatment predictive factor. The observed location-dependent difference in response may reflect anatomical and biological heterogeneity, including variations in ductal obstruction, stromal composition, vascularity, and surgical specimen characteristics. In particular, tumors arising in the pancreatic head frequently cause obstructive jaundice and require biliary drainage during NAC. Moreover, stent dysfunction and cholangitis can lead to treatment delays or interruptions, reducing the delivered dose intensity and potentially attenuating pathological response [26]. PDAC is characterized by a dense desmoplastic stroma and relative hypovascularity, which may limit intratumoral drug penetration; preclinical work has shown that stromal modulation can increase vascular perfusion and enhance gemcitabine delivery [27]. Finally, large-scale molecular profiling studies have demonstrated differences in the genomic and tumor immune microenvironment features between head and body/tail PDAC, suggesting that the anatomic site may also capture intrinsic biological variation relevant to chemosensitivity [28]. Future studies integrating baseline tumor volume, site-stratified analyses, and molecular biomarkers are warranted to delineate the intrinsic predictors of chemotherapy responsiveness in PDAC.

This study has some limitations. First, its retrospective single-center design introduces potential selection bias and center-specific practice patterns. Second, because GS constituted the predominant neoadjuvant regimen in this cohort, the generalizability of the observed response distribution and outcome estimates to other contemporary regimens, such as modified FOLFIRINOX or gemcitabine plus nab-paclitaxel, remains uncertain and warrants external validation. Third, despite efforts toward standardized assessment, interobserver variability remains an inherent limitation of histological grading systems. Although discrepant or ambiguous cases were resolved by consensus review by two pathologists, we did not formally quantify interobserver agreement such as kappa statistics; therefore, broader validation using standardized protocols and reproducibility metrics is warranted. Fourth, the lack of granular information on recurrence patterns and post-recurrence management, including specific salvage regimens and local therapies, limits the mechanistic interpretation of the PRS findings.

In conclusion, histological treatment response evaluated using the JPS grading system provides clinically meaningful prognostic stratification in patients with resectable PDAC treated with NAC and surgical resection, with a Grade ≥ 2 response being associated with better survival and higher R0 resection rates. Importantly, this supports the JPS Grade ≥ 2 cutoff as a practical, viability-oriented pathological endpoint that could enable response-adapted adjuvant decision-making and standardized cross-regimen comparisons in future neoadjuvant trials and real-world cohorts. Therefore, broader adoption of the JPS grading system should be pursued alongside standardized specimen processing and transparent reporting of interobserver reproducibility, to ensure robust interpretability and promote meaningful comparisons across studies.

## Electronic Supplementary Material

Below is the link to the electronic supplementary material.

Supplementary Material 1

## References

[1] Siegel RL, Miller KD, Fuchs HE, Jemal A. Cancer Statistics, 2021. CA Cancer J Clin. 2021;71:7–33. 33433946 10.3322/caac.21654

[2] Hidalgo M. Pancreatic cancer. N Engl J Med. 2010;362:1605–17. 20427809 10.1056/NEJMra0901557

[3] Neoptolemos JP, Palmer DH, Ghaneh P, Psarelli EE, Valle JW, Halloran CM, et al. Comparison of adjuvant gemcitabine and capecitabine with gemcitabine monotherapy in patients with resected pancreatic cancer (ESPAC-4): a multicentre, open-label, randomised, phase 3 trial. Lancet. 2017;389:1011–24. 28129987 10.1016/S0140-6736(16)32409-6

[4] Conroy T, Hammel P, Hebbar M, Ben Abdelghani M, Wei AC, Raoul JL, et al. FOLFIRINOX or Gemcitabine as Adjuvant Therapy for Pancreatic Cancer. N Engl J Med. 2018;379:2395–406. 30575490 10.1056/NEJMoa1809775

[5] Motoi F, Unno M. Adjuvant and neoadjuvant treatment for pancreatic adenocarcinoma. Jpn J Clin Oncol. 2020;50:483–9. 32083290 10.1093/jjco/hyaa018

[6] Unno M, Hata T, Motoi F. Long-term outcome following neoadjuvant therapy for resectable and borderline resectable pancreatic cancer compared to upfront surgery: a meta-analysis of comparative studies by intention-to-treat analysis. Surg Today. 2019;49:295–9. 30877550 10.1007/s00595-019-01786-w

[7] Tempero MA, Malafa MP, Al-Hawary M, Behrman SW, Benson AB, Cardin DB, et al. Pancreatic Adenocarcinoma, Version 2.2021, NCCN Clinical Practice Guidelines in Oncology. J Natl Compr Canc Netw. 2021;19:439–57. 33845462 10.6004/jnccn.2021.0017

[8] Bai X, Li X, Chen Y, Qiao G, Zhang Q, Ma T, et al. Neoadjuvant nab-paclitaxel plus gemcitabine followed by modified FOLFIRINOX for resectable pancreatic cancer: A randomized phase 3 trial. Cancer Cell. 2025;43:2259–67. e2. 41072417 10.1016/j.ccell.2025.09.009

[9] Unno M, Motoi F, Matsuyama Y, Satoi S, Toyama H, Matsumoto I, et al. Neoadjuvant Chemotherapy with Gemcitabine and S-1 versus Upfront Surgery for Resectable Pancreatic Cancer: Results of the Randomized Phase II/III Prep-02/JSAP05 Trial. Ann Surg. 2026;283(1):57–64. 40235447 10.1097/SLA.0000000000006730 PMC12695294

[10] Motoi F, Satoi S, Honda G, Wada K, Shinchi H, Matsumoto I, et al. A single-arm, phase II trial of neoadjuvant gemcitabine and S1 in patients with resectable and borderline resectable pancreatic adenocarcinoma: PREP-01 study. J Gastroenterol. 2019;54:194–203. 30182219 10.1007/s00535-018-1506-7

[11] Wang H, Chetty R, Hosseini M, Allende DS, Esposito I, Matsuda Y, et al. Pathologic Examination of Pancreatic Specimens Resected for Treated Pancreatic Ductal Adenocarcinoma: Recommendations From the Pancreatobiliary Pathology Society. Am J Surg Pathol. 2022;46:754–64. 34889852 10.1097/PAS.0000000000001853 PMC9106848

[12] Evans DB, Rich TA, Byrd DR, Cleary KR, Connelly JH, Levin B, et al. Preoperative chemoradiation and pancreaticoduodenectomy for adenocarcinoma of the pancreas. Arch Surg. 1992;127:1335–9. 1359851 10.1001/archsurg.1992.01420110083017

[13] Chatterjee D, Katz MH, Rashid A, Wang H, Iuga AC, Varadhachary GR, et al. Perineural and intraneural invasion in posttherapy pancreaticoduodenectomy specimens predicts poor prognosis in patients with pancreatic ductal adenocarcinoma. Am J Surg Pathol. 2012;36:409–17. 22301497 10.1097/PAS.0b013e31824104c5 PMC3288807

[14] Chatterjee D, Katz MH, Rashid A, Varadhachary GR, Wolff RA, Wang H, et al. Histologic grading of the extent of residual carcinoma following neoadjuvant chemoradiation in pancreatic ductal adenocarcinoma: a predictor for patient outcome. Cancer. 2012;118:3182–90. 22028089 10.1002/cncr.26651 PMC3269538

[15] van Roessel S, Janssen BV, Soer EC, Farina Sarasqueta A, Verbeke CS, Luchini C, et al. Scoring of tumour response after neoadjuvant therapy in resected pancreatic cancer: systematic review. Br J Surg. 2021;108:119–27. 33711148 10.1093/bjs/znaa031

[16] Tempero MA, Malafa MP, Al-Hawary M, Asbun H, Bain A, Behrman SW, et al. Pancreatic Adenocarcinoma, Version 2.2017, NCCN Clinical Practice Guidelines in Oncology. J Natl Compr Canc Netw. 2017;15:1028–61. 28784865 10.6004/jnccn.2017.0131

[17] Bertero L, Massa F, Metovic J, Zanetti R, Castellano I, Ricardi U, et al. Eighth Edition of the UICC Classification of Malignant Tumours: an overview of the changes in the pathological TNM classification criteria-What has changed and why? Virchows Arch. 2018;472:519–31. 29209757 10.1007/s00428-017-2276-y

[18] Ishida M, Fujii T, Kishiwada M, Shibuya K, Satoi S, Ueno M, et al. Japanese classification of pancreatic carcinoma by the Japan Pancreas Society: Eighth edition. J Hepatobiliary Pancreat Sci. 2024;31:755–68. 39074998 10.1002/jhbp.12056 PMC11589393

[19] Chetty R, Gill P, Govender D, Bateman A, Chang HJ, Deshpande V, et al. International study group on rectal cancer regression grading: interobserver variability with commonly used regression grading systems. Hum Pathol. 2012;43:1917–23. 22575264 10.1016/j.humpath.2012.01.020

[20] Matsuda Y, Ohkubo S, Nakano-Narusawa Y, Fukumura Y, Hirabayashi K, Yamaguchi H, et al. Objective assessment of tumor regression in post-neoadjuvant therapy resections for pancreatic ductal adenocarcinoma: comparison of multiple tumor regression grading systems. Sci Rep. 2020;10:18278. 33106543 10.1038/s41598-020-74067-z PMC7588464

[21] Mori S, Aoki T, Sakuraoka Y, Shimizu T, Yamaguchi T, Park KH, et al. Predictors of Poor Pathological Response to Neoadjuvant Gemcitabine Plus S-1 Chemotherapy in Patients With Pancreatic Ductal Adenocarcinoma. Pancreas. 2021;50:744–50. 34016892 10.1097/MPA.0000000000001826

[22] Truty MJ, Kendrick ML, Nagorney DM, Smoot RL, Cleary SP, Graham RP, et al. Factors Predicting Response, Perioperative Outcomes, and Survival Following Total Neoadjuvant Therapy for Borderline/Locally Advanced Pancreatic Cancer. Ann Surg. 2021;273:341–9. 30946090 10.1097/SLA.0000000000003284

[23] He J, Blair AB, Groot VP, Javed AA, Burkhart RA, Gemenetzis G, et al. Is a Pathological Complete Response Following Neoadjuvant Chemoradiation Associated With Prolonged Survival in Patients With Pancreatic Cancer? Ann Surg. 2018;268:1–8. 29334562 10.1097/SLA.0000000000002672 PMC6178802

[24] Kim SS, Ko AH, Nakakura EK, Wang ZJ, Corvera CU, Harris HW, et al. Comparison of Tumor Regression Grading of Residual Pancreatic Ductal Adenocarcinoma Following Neoadjuvant Chemotherapy Without Radiation: Would Fewer Tier-Stratification Be Favorable Toward Standardization? Am J Surg Pathol. 2019;43:334–40. 30211728 10.1097/PAS.0000000000001152

[25] Janssen BV, Tutucu F, van Roessel S, Adsay V, Basturk O, Campbell F, et al. Amsterdam International Consensus Meeting: tumor response scoring in the pathology assessment of resected pancreatic cancer after neoadjuvant therapy. Mod Pathol. 2021;34:4–12. 33041332 10.1038/s41379-020-00683-9

[26] Vehvilainen S, Kuuliala A, Udd M, Nurmi A, Peltola K, Haglund C, et al. Cholangitis and Interruptions of Neoadjuvant Chemotherapy Associate with Reduced Overall and Progression-Free Survival in Pancreatic Cancer. Ann Surg Oncol. 2024;31:2621–31. 38153645 10.1245/s10434-023-14793-6 PMC10908635

[27] Olive KP, Jacobetz MA, Davidson CJ, Gopinathan A, McIntyre D, Honess D, et al. Inhibition of Hedgehog signaling enhances delivery of chemotherapy in a mouse model of pancreatic cancer. Science. 2009;324:1457–61. 19460966 10.1126/science.1171362 PMC2998180

[28] Abdelrahim M, Esmail A, Kasi A, Esnaola NF, Xiu J, Baca Y, et al. Comparative molecular profiling of pancreatic ductal adenocarcinoma of the head versus body and tail. NPJ Precis Oncol. 2024;8:85. 38582894 10.1038/s41698-024-00571-4 PMC10998911

